# Mr. Smart's Case of Inflammation after Vaccination

**Published:** 1807-02

**Authors:** Thomas Smartt

**Affiliations:** Union Street, Bishopsgate


### *To the Editors of the Medical and Phyſical Journal.*

GENTLEMEN,

THROUGH the medium of your very useful publication I make the medical world acquainted with a very uncommon and distressful disease, which occurred in a patient of mine, whom I had inoculated with vaccine matter.

The subject in question was a very healthy child, four months old; the matter inserted in the arm I took from a child between two and three years old, apparently of sound constitution and free from any eruptive disease, who had been inoculated seven days before, by Mr. Weston of Shoreditch; the pustule was well formed, with a small areola.

The progress of the disease in my patient promised well until the sixth day, when calling to take matter for future inoculation, I found the pustule had been violently squeezed and broken down. The seventh day some lymph remaining on one side, four lancets were moistened with it; but owing to the accident to the pustule, I cautioned the gentleman for whom I moistened three of them, that it ought not to be depended on.

Instead of the circumscribed areola characteristic of this disease, the arm was much inflamed and tumid, the pustule was of a lead colour. The eighth day the arm was inflamed to the shoulder and wrist. The tenth the hand participated of it, and it had spread over the right breast. In this way every day the disease crept along, until on the 19th day, the whole of the abdomen, left arm, back and loins, were inflamed. About the 15th day, the inoculated arm became pallid, the cuticle appeared somewhat shrivelled, and it remained considerably enlarged, but the swelling was too firm to receive easily the impression of the finger. The 22d day the inflammation had reached the feet, but at this late period of the disease it did not put on so florid an appearance as that which attacked the superior parts of the trunk and arm. The 26th day, the hands, arms, legs, and feet remained considerably swollen and tender, and the cuticle peeled off from the affected parts. On the 27th day, a slight degree of the inflammation made its appearance at the back of the neck, and on the integuments covering the os occipitis. The 28th day, the disease had not spread any farther. The 29th day, the abdomen abdomen became considerably swollen and tense, and at night the child died.

In like manner as the inflammation had quitted the inoculated arm, so did it every other part in the order in which it had attacked them; thus, when it had arrived at the thighs, the chest and upper part of the back were pretty well free, but there was once or twice a slight return on the arm and breast. The body as well as the extremities remained swollen, and put on a pallid appearance, and was evidently anasarcous.

The ulcer on the arm was completely cicatrized about the 26th day; it was first dressed with ung. hyd. nitrat. afterwards poulticed for three days, and lastly dressed with cerat. lap. calam. occasionally touching it with lunar caustic; it was never deep or foul, but for the greater part of the time put on an healthy appearance.

The arm was at first wrapped in cloths wet with aq. lith. acet. comp.; and two or three poultices, made with it and crumb of bread, were applied over the chest and belly; but finding the cuticle very suddenly peel off, I immediately discontinued all saturnine applications, and used afterwards a wash of acetum spt. vin. each one part, aq. puræ, six parts. However, no apparent good was derived from any application.

The child's health during the whole of this process was, as may be expected, considerably deranged. The bowels were much out of sorts, and the breathing very much impeded, more particularly when the chest was the seat of the inflammation. In the early stage of the disease the child was much inclined to sleep, but afterwards it became very restless and uneasy throughout. From the beginning the pulse was very quick, and the skin extremely hot, but there were no vesications.

The medical treatment was of the antiphlogistic kind, with occasional purges, until within a day or two of the catastrophe, when it became necessary to have recourse to wine whey and cordials.

During the progress of the disease my anxiety was of course much excited, as I had heard but of one similar instance, and which is I believe reported in a former number of this work; I therefore took every opportunity of enquiring amongst practitioners most likely to be well informed on the subject of vaccination, but I was not able to hear of more than two or three cases which bore any similarity, and one of these was inoculated with matter from my patient. In this child the punctured part became evidently evidently inflamed in eight hours after inoculation, and the inflammation increased rapidly over the arm and whole of the body; but although the child was expected to die, it did at length recover.

Another case of a similar nature occurred in a child about six months old; the vaccine disease in this may be said to have been compleat, as the inflammation did not make its appearance until the 16th day, at which time the eschar on the arm was actually dropping off. In this child the disease first shewed itself behind one of the ears, from whence it spread down the neck, across the breast and back, and so on progressively over the whole of the body and lower extremities, but did not attack the inoculated arm until a considerable number of days had elapsed.

*Union Street, Bishopsgate,* I am, &c.

*Sept.* 14, 1806. THOMAS SMARTT.*

* We wish Mr. S. had been more minute in his account of the cause, manner, and degree of injury inflicted on the vesicle, as we have no doubt that this was the exciting cause of all the subsequent mischief. ED.

